# Midterm clinical outcomes of collateral ligament repair of the thumb and lesser digits: a retrospective analysis of 35 cases

**DOI:** 10.1186/s12891-022-05605-1

**Published:** 2022-07-22

**Authors:** Mehmet Sukru Sahin

**Affiliations:** Department of Orthopaedics and Traumatology, Baskent University Alanya Research and Practice Center, Saray Mahallesi Yunus Emre Caddesi No:1, 07400 Alanya, Antalya Turkey

**Keywords:** Finger injury, Proximal interphalangeal (PIP) joint collateral ligament injury, Thumb collateral ligament injury, Collateral ligament repair, Suture anchor

## Abstract

**Background:**

Finger collateral ligament injuries are common, and conservative treatment usually works well. However, complete ruptures that lead to instability could cause painful disability. This study presents our clinical experience and a qualitative functional evaluation following the surgical repair of the thumb and proximal interphalangeal (PIP) joint collateral ligament ruptures.

**Methods:**

Thirty-five patients (22 men and 13 women), diagnosed via a physical examination and magnetic resonance imaging (MRI) with a total collateral ligament rupture in the metacarpophalangeal thumb (16) and PIP joints of the lesser digits (19) and treated surgically, were evaluated retrospectively. The limited range of motion; functional score by Saetta; disabilities of the arm, shoulder, and hand (DASH) score; pre- and post-operative pain, deformity level; and post-operative ability to grip keys, buttons, and jars were measured. The significance of the change between the pre and post-operative visual analog scale for pain (VAS) scores were evaluated using the Wilcoxon signed-rank test. The difference between the lesser digits and thumb groups by patient age was evaluated using the Mann–Whitney-U test. All data, such as the mean, range, and standard deviation, were calculated using SPSS.

**Results:**

The mean pre- and post-operative VAS scores were 4.8 (from 3 to 7) and 0.91 (0 to 4), respectively. The mean post-operative limitation in the range of motion was 9.78° (*s* = 14.47) for lesser digits and 6.87° (*s* = 12.29) for the thumb. According to Seatta et al., the final functional score was 62.5% excellent, 25% good, and 12.5% moderate for the thumb and 84.2% excellent, 10.5% good, and 5.3% poor for the lesser digits. The mean post-operative DASH score was 13.55 (SD: 8.77) for lesser digits and 14.22 (SD: 8.9) for the thumb. The mean contralateral (healthy) hand DASH score was 0.75 (SD: 1.05) for lesser digits and 0.75 (SD: 1.05) for the thumb. For the thumb and lesser digits, the *z*-scores were − 3.55 and − 3.787, respectively, and the progress of the VAS score was significant (*p* < 0.05).

**Conclusion:**

After a 40-month follow-up for 35 acute, subacute, and chronic cases, the results suggest that direct and suture-anchor repairs are feasible, painless treatments associated with good finger function.

## Introduction

In sports or at work, injuries to the collateral ligament of the thumb’s metacarpophalangeal (MCP) joint and the proximal interphalangeal (PIP) joints of the fingers are common [1]. The most common mechanism for a thumb ulnar collateral ligament (UCL) rupture is the forced abduction and hyperextension of the MCP joint of the thumb [2–7], which can occur when someone falls on the thumb or the thumb is struck, forcing it into abduction. A similar mechanism occurs when a ski pole is caught in the ground, and the thumb is forced into the pole handle by the skier’s momentum. This type of injury is also known as skier’s thumb and most commonly occurs in skier falls [2]. Chronic injuries caused by ligament attenuation under repetitive trauma are called Gamekeeper’s thumb [2].

A physical examination with varus and valgus stress tests on the joint is useful for diagnosis. In individuals with an ambiguous clinical assessment, stress radiographs may be taken to determine the degree of laxity better [8]. In conjunction with a clinical examination, ultrasonography is a noninvasive and cost-effective method for assessing thumb collateral tears [8]. In addition, magnetic resonance imaging (MRI), especially with specific extremity coils, is less cost-effective but more consistent in facilitating diagnosing collateral thumb injuries [8]. However, MRI is less useful for the PIP joint because of its small size.

Splinting or immobilization for three weeks is the standard therapy for partial ruptures [9]. However, in injuries with a total ligament tear, such conservative treatment frequently results in discomfort, instability, and pinch strength loss [9]. In patients who are manual laborers or participate in sports, the primary repair of the collateral ligament of the thumb MCP or finger PIP joints is frequently suggested [9, 10].

Many surgical techniques for repairing or reconstructing acute and chronic unstable collateral injuries of the thumb and fingers have been described. After the innovation of the suture anchor, the repair technique was revolutionized, and the number of publications regarding the clinical follow-up results increased tremendously. However, most of the literature deals with the results of thumb lateral ligament tears. Very few reports on the clinical follow-up results of PIP joint collateral ligament repair or the results of PIP and thumb collateral ligament repair are available. The literature lacks large cohorts with long follow-up periods. This study contributes to the literature by reporting the follow-up results on the collateral repair of the thumb and PIP joint, either with suture anchor reinforcement or direct repair. This study presents our clinical experience and a qualitative functional evaluation following the surgical repair of the thumb and PIP joint collateral ligament ruptures.

### Materials and methods

This retrospective study was approved by the ethics committee of our institution and was registered under the protocol. The present study was conducted following a recognized international standard, including the principles of the Declaration of Helsinki.

Thirty-seven patients (24 men and 13 women) diagnosed via a physical examination and MRI between 2012 and 2020 with a total collateral ligament rupture in the MCP joint thumb (16) and PIP joints of the lesser digits (21) and treated surgically were evaluated retrospectively. Two patients (men with PIP collateral ligament injuries) were missed and excluded from the study. Informed consent was obtained from all patients before enrollment in the study.

The clinical decision regarding a collateral ligament rupture of the PIP joint of the lesser digits was made with the joint extended and 30° flexion, as described by Minamikawa et al. [11]. The authors suggested that a lateral angulation of 10° in extension and 20° of lateral angulation at 30° of flexion indicated complete collateral ligament rupture. In addition, the absence of an endpoint during stress loading was a contributing factor.

The thumb was examined for ligamentous laxity using fluoroscopy under local anesthesia by a senior surgeon in the radiology department. The author determined that a total UCL rupture was present when an opening > 35° of the MCP joint was assessed at 0° and 30° of flexion or when a > 10° difference in the joint opening of the MCP compared with the contralateral thumb [5].

After clinical examination, MRI was used to verify and support the diagnosis. The patients’ pain scores were recorded using the VAS for pain. The functional score for the fingers was evaluated using scale criteria described by Seatta et al. [12] and the disabilities of the arm, shoulder, and hand (DASH) score. The range of motion of the fingers was recorded in degrees with a finger goniometer. (™: Baseline Finger Goniometers, White Plains, NY, USA).

### Inclusion and exclusion criteria

Injuries were diagnosed via a physical examination and confirmed with an MRI scan as a collateral ligament total rupture of the thumb or PIP joint. Excluded cases include: missed patients on follow up or injuries to the collateral ligament with multiple associated traumas or wrist and metacarpal fractures, injuries in the collateral ligament of the lesser digits’ MCP joint and fingers that do not meet the criteria by Minamikawa et al. [11] for total rupture, and open injuries. According to these criteria, two patients with PIP collateral injuries were excluded from the study.

### Statistical methods

The significance of the change between the preoperative and post-operative VAS scores was evaluated using the Wilcoxon signed-rank test. The difference between the lesser digits and thumb groups regarding patient age was evaluated using the Mann–Whitney-U test. All data, such as the mean, range, and standard deviation, were calculated using SPSS.

### Operative technique

**Fig. 1:**
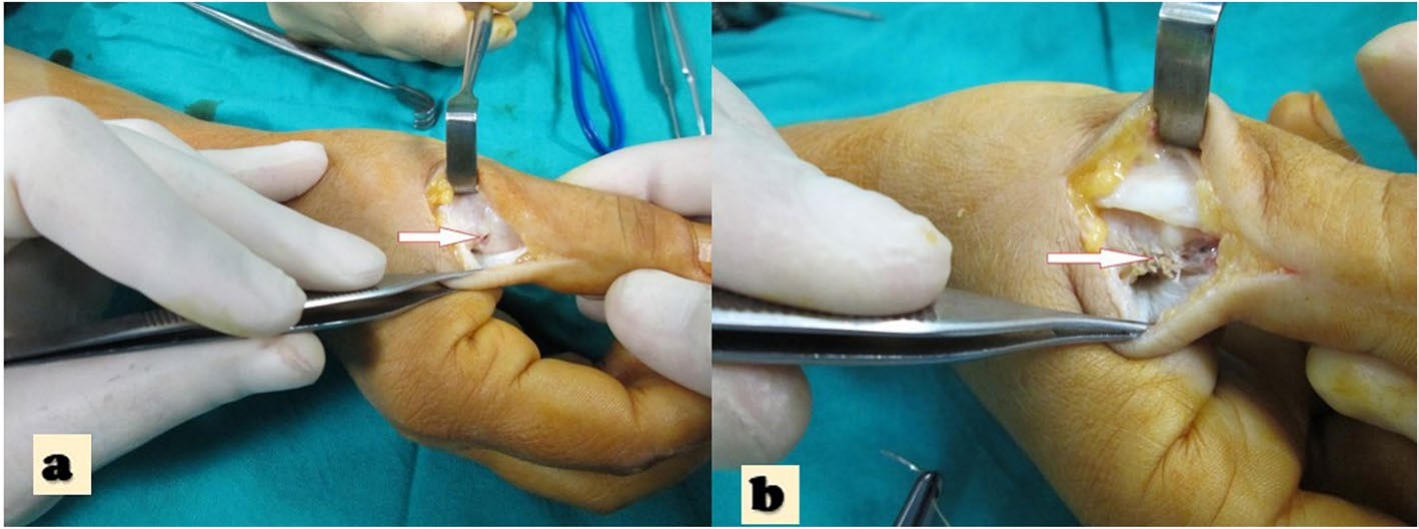
**a** The intraoperative photo, notice the White arrow that demonstrates the thumb UCL rupture**. b.** white arrow demonstrates the stiches at repair side that represent direct repair

**Fig. 2:**
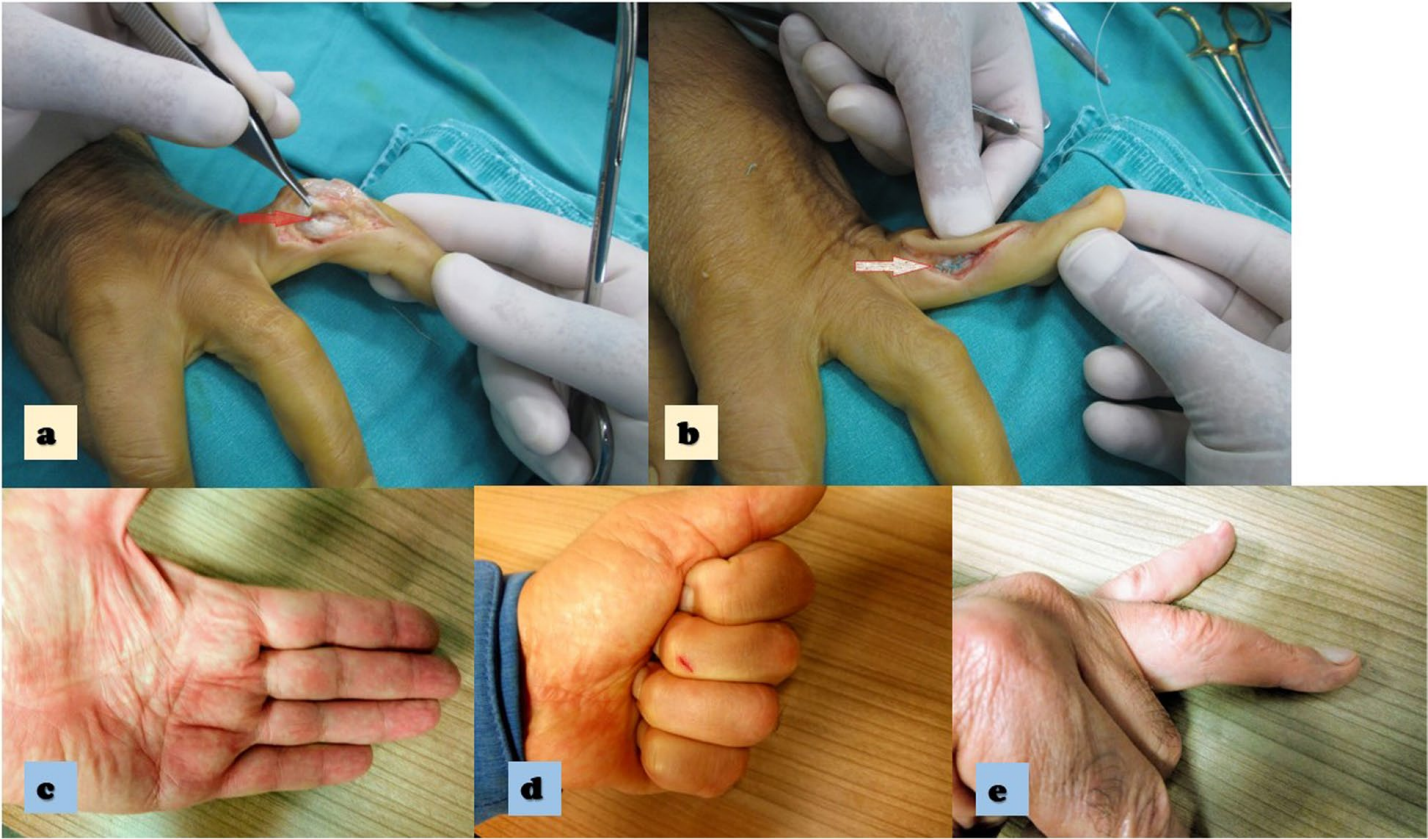
**a** The intraoperative photo, notice the red arrow that demonstrates the 4.th finger RCL rupture. **b** white arrow demonstrates the stiches at repair side that represent direct repair. **c** The post operative photo at 1. Year follow up, demostrates the full extension. **d** The post operative photo at 1. Year follow up, demostrates the full felxion of the.^4th^ finger. **e** The post operative l photo at 1. Year follow up, demostrates the finger appearence

The patients were operated on by a single surgeon using the same approach. The radial-ulnar-sided approach was used with a 2-cm longitudinal incision for the thumb (see Fig. 1). The dorsolateral approach to the PIP joint was used for lesser digits, and a wide-based V-shape incision was made (see Fig. 2). The skin was retracted dorsally as a full-thickness flap.

**Fig. 3:**
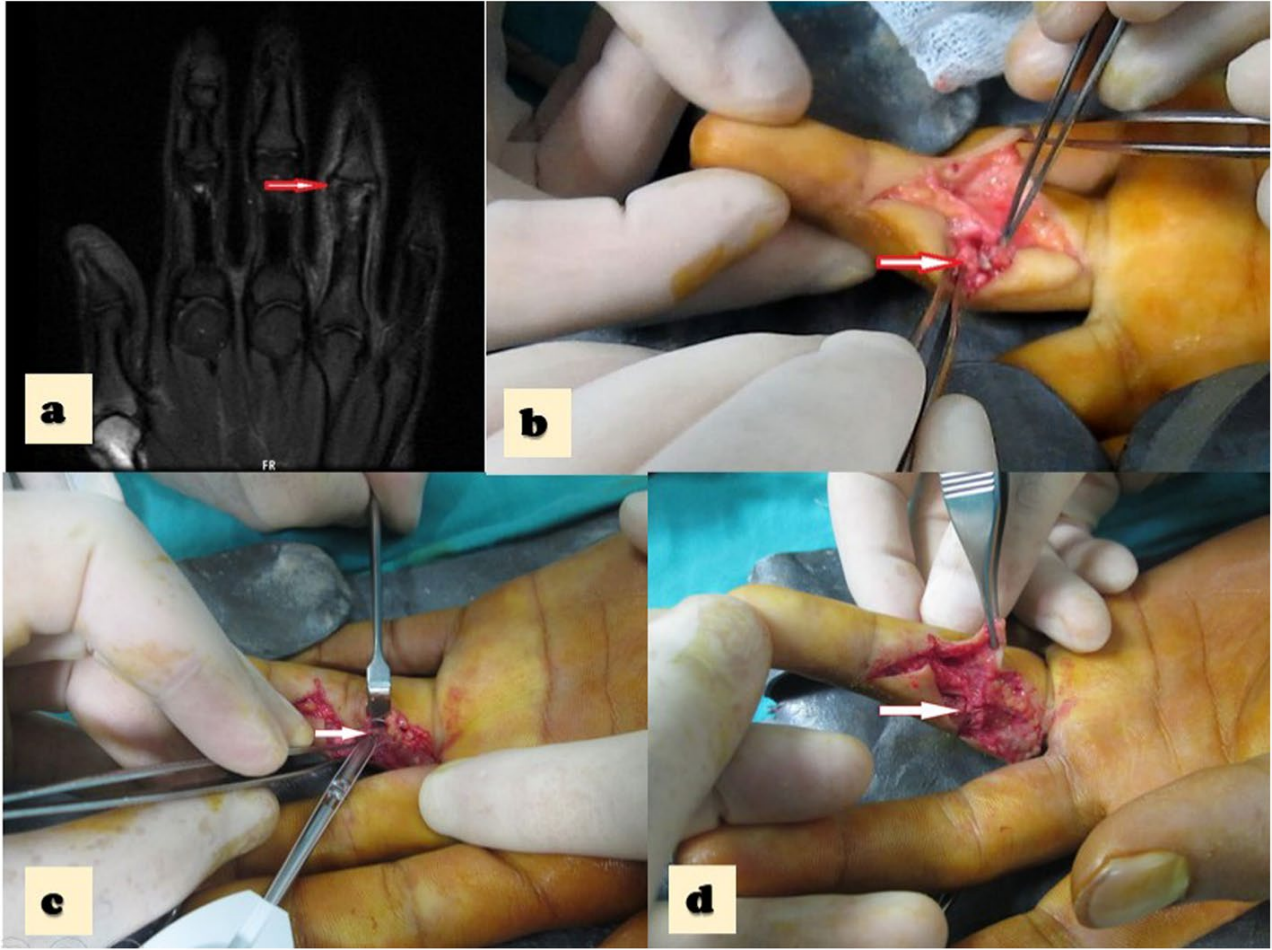
**a** The pre operative MRI that demonstrates the forth finger RCL rupture. **b** The intraoperative photo, notice the arrow that demonstrates the 4.^th^ finger RCL rupture. **c** The intraoperative photo notice the white arrow that demostrates the suture anchore augmented repair. **d** The intraoperative photo notice the white arrow that demostrates the completed repair side

The extensor mechanism and capsule were incised, and the ruptured collateral ligament was repaired. Collateral ligament injuries were observed in almost all cases, and the rupture location was at the origin or insertion point of the ligaments. A mini corkscrew (suture anchor) was used for fixation (1.3 De-Puy Mitek, Johnson & Johnson Company, England; see Fig. 3). The extensor mechanism and capsule were repaired, and the skin was closed with mattress sutures.

In two cases in the thumb group, admitted extremely late (after 395 and 210 days), the author performed MCP joint capsulorrhaphy with adductor aponeurosis advancement to the proximal phalanx, referring to the study by Neviaser et al. [13]. In the late-admission case (after 150 days) with a tear of the fifth finger PIP radial collateral ligament (RCL), the author augmented the lateral band of the extensor apparatus or used the oblique retinacular ligament to repair the collateral ligament (referring to the study by Littler and Colley [14]). In cases with bony fragments, the reduction and *k*-wire fixation were achieved. Later, the primary or suture anchor augmented repair was used. In patients with a volar plate injury, the collateral ligament and volar plate repair was performed using a direct method or suture anchor.

After operation, the group with the UCL/RCL repair of the thumb (2 weeks) was immobilized in a short-arm thumb spica cast, leaving the interphalangeal joint free and keeping the MCP joint in a 10° to 20° flexion and adduction/abduction. The lesser digit group was immobilized in a custom orthotic in 20–30 degree flexion, and the patient was later sent to a hand physiotherapist.

## Results

In this study, 35 patients who had undergone surgery for thumb and PIP joint collateral ligament repair were identified: 22 (62.9%) were male, and 13 (37.1%) were female. The mean age at presentation was 40.6 years (ranging from 18 to 65 years). Regarding the patient age, both groups demonstrated no statistically significant difference (Mann–Whitney-U test, *p* > 0.05). The mean post-operative follow-up time was 40.44 (from 8 to 87) months for the thumb and 40.74 (from 5 to 99) months for the lesser digits.

The rate of ability to grip keys, buttons, and jars postoperatively was 93.8% and 94.7% for the thumb and lesser digits, respectively.

The mean pre- and post-operative VAS scores were 4.75 (from 3 to 7) and 1.56 (from 0 to 3), respectively, for the thumb. The mean pre- and post-operative VAS scores for the lesser digit were 4.84 (from 4 to 6) and 0.84 (from 0 to 4), respectively. The z-score for the thumb was − 3.55 and for the lesser digits was − 3.787, and the progress of the VAS score for the thumb and lesser digits was significant (*p* < 0.05; see Table 1). The mean post-operative limitation of the range of motion was 9.78° (*s* = 14.47) for lesser digits and 6.87° (*s* = 12.29) for the thumb.

**Table 1:** The statistics^a^ that depicts the improvement of visual analog scale after the treatment

Fingers		post_VAS—pre_VAS	
Thumb	Z	-3.550^b^	*P* < 0.005 (0,00,038)
	^a^Asymp. Sig. (2-tailed)	0,002	significant
Lesser digits	Z	-3.787^b^	*P* < 0.005 (0,00,015)
	^a^Asymp. Sig. (2-tailed)	<0,001	significant

The improvement of pain (VAS) after the surgical repair was found to be statistically significant for both thumb and lesser digits (*p* < 0.005)

^a^Wilcoxon Signed Ranks Test

^b^Based on positive ranks

According to Seatta et al. [12], the final functional scores were 62.5% excellent, 25% good, and 12.5% moderate for the thumb and 84.2% excellent, 10.5% good, and 5.3% poor for the lesser digits. In addition, the mean post-operative DASH score for lesser digits was 13.55 (SD: 8.77) for lesser digits and 14.22 (SD:8.9) for the thumb. The mean contralateral (healthy) hand DASH score was 0.75 (SD: 1.05) for lesser digits and 0.75 (SD: 1.05) for the thumb.

**Fig. 4:**
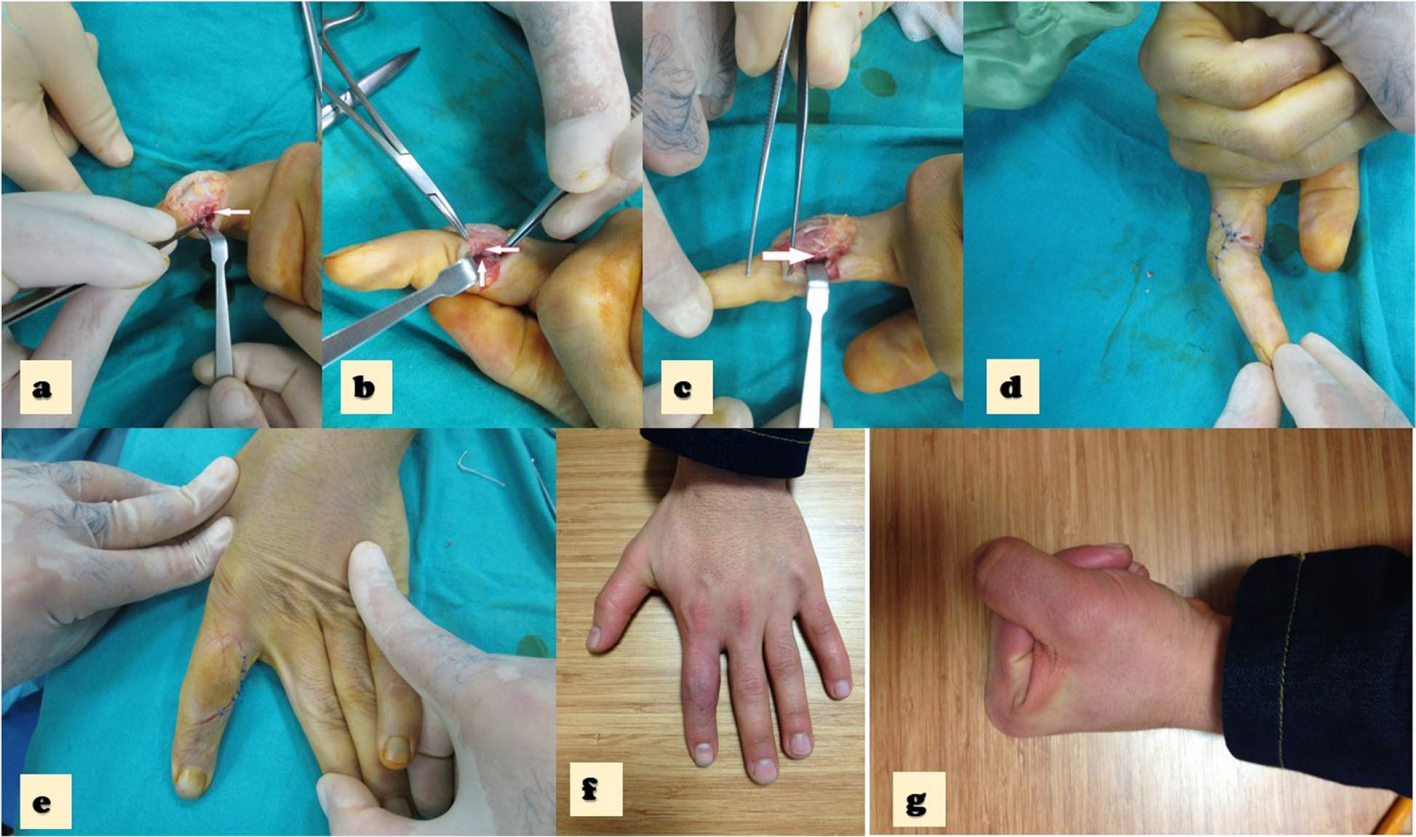
**a** The intraoperative photo, notice the white arrow that demonstrates the 2^th^ finger UCL rupture. **b** The intraoperative photo, notice the rwhite arrow that demonstrates the 2^th^ finger UCL rupture. **c**. White arrow demonstrates the stiches at repair side that represent direct repair. **d** The Intra operative photo demostrates the appearence of the finger after completing the repair. **e** The Intra operative photo demostrates the appearence of the finger after completing the repair. **f** The post operative photo at 1. year follow up, demostrates the full extension with mild deformity of the 2^th^ finger. **g** The post operative l photo at 1. year follow up, demostrates mild flexion limitation of the 2.^th^ finger

Postoperatively, the ratio of deformity of the lesser digits was 57.9% without deformity, 36.8% minimal (fusiform edematous appearance of the PIP joint; see Fig. 4), and 5.3% moderate. Similarly, the post-operative ratio of deformity of the thumbs presented 75% no deformity and 25% minimal deformity (fusiform edematous appearance of the MCP joint).

## Discussion

In the acute, subacute, and chronic settings of the 35 cases, the author demonstrated that direct repair and suture anchor-augmented repair resulted in good function and no pain after a follow-up of 40 months. The direct or suture anchor-augmented methods were used in the acute and subacute cases. However, different methods were used in two cases from the thumb group and one case from the lesser digit group. The method for late thumb UCL repair (see the surgical technique section) was cited by Neviaser et al. [13], and several surgeons in various journals have also cited the described technique [15, 16].

There is no consensus on the treatment of these injuries. Specific surgical indications have been described, such as Stener-type lesions, in which the collateral ligament becomes caught between the lateral band and central slip [17–19], irreducible dislocations [20, 21], and recurrent subluxation [22]. Some authors have advised surgical treatment for full-thickness collateral ligament ruptures [16, 23, 24], whereas others have urged surgical treatment primarily for manual laborers and professional athletes, referring to superior pain relief and a fast return to function [25–27]. Others have proposed nonoperative care, noting post-surgery stiffness as the “biggest barrier to treatment” [28, 29]. These suggestions are predominantly based on single-center case studies (with a limited number of patients) [30]. Compared to the literature, the present study had a good number of cases and a significant follow-up time.

Ali et al. (1984) compared conservative therapy and surgical repair of patients with collateral ligament injuries of the PIP joint [10]. They discovered that surgical treatment offered superior results in those years. Unfortunately, it will take another 15 years for the first report on using suture anchors to repair the collateral ligament of the PIP joint.

Most reports in the literature concern thumb UCL rupture repair [12, 31–36] (see Table 2). Furthermore, two author groups reported on the thumb RCL alone or with UCL repair [37, 38] (see Table 2). Moreover, regarding PIP joint collateral ligament repair, few cohorts are reported in the literature [9, 10, 39, 40] (see Table 2).

**Table 2:** Classification of papers regarding thumb, lesser digits, and setting to chronic, acute

		**Thumb**			**Thumb+Lesser digits **		**Lesser digits **
		UCL	RCL	UCL+RCL			MCP+ PIP
1	Christensen T et al. Hand (N Y). 2016 [32]	Chronic Thumb UCL					
2	Kara et al. Acta Chir Orthop Traumatol Cech. 2019 [36]	Acute Thumb UCL					
3	Catalano LW 3rd et al. J Hand Surg Am. 2006 [38]		Chronic RCL Thumb				
4	Chuter et al. Injury.2009. [33]	Acute UCL thumb					
5	Glickel et al. J Hand Surg Am. 1993 [31]	Chronic UCL Thumb					
6	Gvozdenovic and Boeckstyns. Tech Hand Up Extrem Surg. 2014 [37]			**Chronic 4 RCL +14UCL Thumb**			
7	Katolik et al. Plast Reconstr Surg. 2008 [34]	Acute UCL thumb.					
8	Moharram AN. Ann Plast Surg. 2013 [35]	Acute or Delayed cases. 27 UCL Thumb					
9	Saetta et al. J Hand Surg Br. 1992 [12]	Acute 25 UCL Thumb.					
10	Lee et al. Acta Orthop Traumatol Turc. 2017 [40]						Seven patient non-op and 10 patient PIP collateral lig repair.
11	Waxweiler C et al. Plast Reconstr Surg. 2019 [42]						Acute and Chronic 46 MCP lesser digits.
12	Vigasio A et al. Tech Hand Up Extrem Surg. 2012 [24]						MCP lesser digit
13	Lee et al. J Hand Surg Eur Vol. 2012 [1]				Chronic 7-UCL+1 RCL thumb and 3 PIP		
14	Tuncay and Ege Croat Med J. 2001 [41]				Chronic; 6 Thumbs MCP -2 fifth finger PIP )		
15	Kato H et al. J Hand Surg Br. 1999 [9]				Acute, 7 Thumb and 11 PİP joint.		
16	Kato et al. Scand J Plast Reconstr Surg Hand Surg. 2003 [39]						Not determined. PİP joint collateral ligament.
17	Ali MS. J Hand Surg Br. 1984 [10]						Acute. PİP joint
18	Wong JC et al. Hand (N Y). 2014 [43]						Subacute-to ChronicLesser digits’MCP collateteral.
19	Current Report	**Acute +subacute and chronic cases.**			35 patients.19 Lesser digit PİP collarela ligament + 16 Thumb MCP collateral ligaments		

Bui et al. (2021) reported a systematic review of the literature concerning studies reporting the clinical results of the surgical repair of acute complete collateral ligament ruptures of PIP joints [30]. They detected four studies reporting on 68 patients with acute ligament injuries of the PIP joint, 47 of whom underwent acute surgical repair [9, 10, 39, 40]. Based on this systematic literature review, the surgical repair of an acute PIP joint collateral ligament rupture is feasible, although the published literature has both quantitative and qualitative flaws [30]. Compared with the existing literature regarding the clinical follow-up results of PIP joint collateral ligament rupture repair, our cohorts have the largest number of patients and longest follow-up period.

Saetta et al. (1992) assessed 25 patients following the repair of an acutely ruptured UCL of the MCP thumb joint [12]. They compared two suture materials. The first was a horizontal mattress suture using a 4/0 polybutylate-coated braided suture (nine patients), and the second method involved repairing with a prefashioned steel wire (16 patients). They concluded (after a mean 12.9-month follow-up) that both techniques were equally effective, and using the more expensive steel wire, while technically satisfying and easy to perform, offers no clinical advantage over the simple suture.

Glickel et al. (1993) described a technique of ligament (UCL of the MCP joint of the thumb) replacement for 26 chronic cases [31]. They used a free tendon graft inserted through two holes at the base of the proximal phalanx and a single transverse hole in the metacarpal neck. The follow-up period averaged 4.5 years. They reported excellent results in 20 patients, good results in four, and fair results in two.

Tuncay and Ege (2001) presented a series of patients in whom the Statak® suture anchor was used to repair the finger joints’ chronically unstable collateral ligament injuries [41]. There were eight fingers (six thumbs and two fifth fingers) with a mean follow-up of 19 months without additional reinforcement. They concluded that using the suture anchors for repairing chronic ruptures of the collateral ligaments of fingers is an effective method with excellent clinical results. However, their cohort was small and with a shorter follow-up time than the present study.

Waxweiler et al. (2019) studied 46 patients. Christensen et al. (2016) studied 12 patients, and Wong et al. (2014) studied 19 patients. All postulated that repairing a chronic UCL or lesser digits’ MCP collateral ligament injury with locally available tissue seems a reasonable alternative to ligament reconstruction [32, 42, 43]. Furthermore, Christensen et al. [32] performed the longest follow-up in the literature at 15 years. According to the current study’s findings, the suture anchor offered successful repair and allowed us to perform the repair in chronic cases (except for three very late admissions).

Catalano et al. (2006) compared two techniques involving direct repair and reconstruction with palmaris longus in acute grade III tears of thumb RCL [38]. The mean follow-up period was 59 months, and they did not report a statistically significant difference between the two methods regarding grip and pinch strength or stability. In the current report, the author performed the direct repair in acute and subacute admissions. Additionally, in chronic cases, the author performed MCP joint capsulorrhaphy with adductor aponeurosis advancement to the proximal phalanx, referring to the study by Neviaser et al. [13].

Chuter et al. (2009) reported the results of 127 patients who were acutely injured and surgically treated, the largest cohort in the current literature [33]. They repaired the ruptured UCL with absorbable 2/0 braided sutures without using a suture anchor and reported satisfactory results with a low rate of long-term complications.

Gvozdenovic and Boeckstyns (2014) described a new technique for reconstructing chronic lesions of the collateral ligaments of the MCP ligaments of the thumb, using a Bio-Tenodesis screw for the fixation of a tendon graft in a triangular manner with the proximal apex and allowing early mobilization, starting two weeks after the operation [37]. The authors used this technique and reported a review of a consecutive series of 18 patients, and the mean follow-up period was 26 months. Their technique offers a short rehabilitation period with good functional results.

Katolik et al. (2008) compared two 30-patient cohorts who had a complete rupture of the UCL of the MCP joint of the thumb [34]. The first group was treated with an intraosseous suture anchor followed by early mobilization, and the second group was treated with a pull-out suture tied over a button with cast immobilization. The mean follow-up time was 29 months. They concluded that both repair procedures were safe and effective for treating thumb UCL injuries.

Lee et al. (2012) described a surgical technique for anatomical collateral ligament reconstruction using a free tendon graft and intraosseous suture anchors and presented their clinical outcomes [1]. Eleven patients who underwent collateral ligament reconstruction at the PIP or MCP joints were enrolled in their study. There were eight collateral ligaments (either UCL or RCL) of the thumb and three collateral ligament PIP joints (11 patients). They reported excellent results for 10 patients and good clinical results in one patient. The mean follow-up time was 9.2 months.

Moharram et al. (2013) prospectively evaluated the functional results of 27 patients who underwent open repair of the UCL of the thumb using either acutely or delayed micro-anchors [35]. After a 16-month follow-up, they reported good-to-excellent results regarding stability, safety, and pain.

Parallel to Ali et al. [10], after 33 years, Lee et al. [40] compared the clinical outcomes of the surgical treatment of 10 patients with conservative treatment of collateral ligament tears of the PIP joint. The mean follow-up time was 6 months. In the surgical group, they used a suture anchor. They found that patients who underwent surgical repair reported less pain and had a PIP joint with a better appearance at the final follow-up than those who did not undergo surgical repair.

**Fig. 5:**
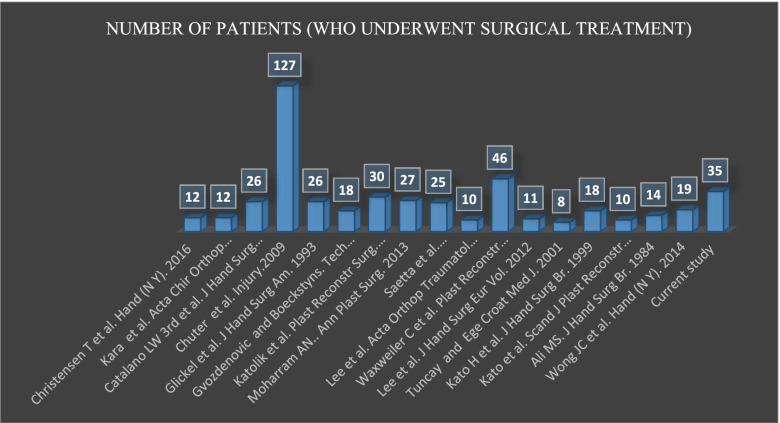
List of publications on number of patients

**Fig. 6:**
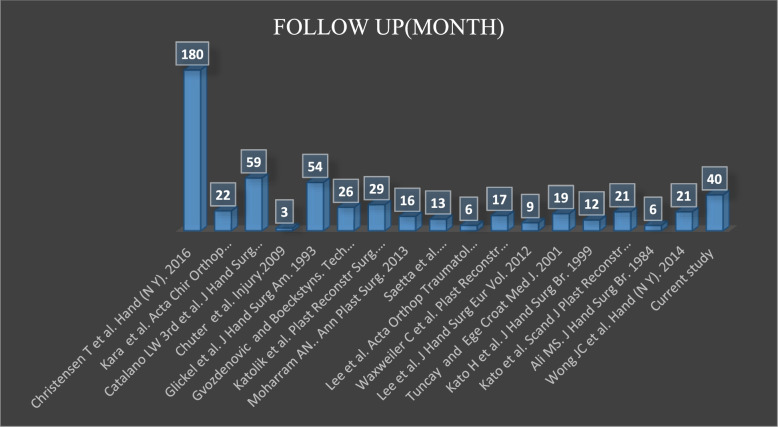
List of publications on follow up time

Lee et al. [1] and Moharram et al. [35] studied smaller cohorts with shorter follow-up times than those in the current study. Only three groups of authors [1, 9, 41] reported clinical results on thumb and lesser digits collateral ligament repair (MCP and PIP joints). Regarding the number of patients and the follow-up period, the current study has strengths compared to the recent literature (see Figs. 5 and 6).

The major limitation of this study is the lack of a comparison group and its retrospective nature. Future prospective randomized controlled trials would be more informative and contribute more comprehensive knowledge to the literature.

## Conclusion

The author believes that hand surgeons might choose a surgical treatment option for collateral ligament rupture of the fingers. After a mean follow-up of 40 months in 35 acute, subacute, and chronic cases, the results of this study suggest that direct and suture-anchor repairs are feasible, painless, and associated with good finger function.

## Data Availability

All data generated or analyzed during this study are included in this published article and its supplementary information files.

## Abbreviations

PIP: Proximal interphalangeal
MCP: Metacarpophalangeal
UCL: Ulnar collateral ligament
RCL: Radial collateral ligament
VAS: Visual analog scale
MRI: Magnetic resonance imaging
DASH: Disabilities of the arm, shoulder, and hand
